# Posterior Shoulder Instability: Spectrum of Presentation and Treatment Outcomes in a Case Series

**DOI:** 10.7759/cureus.96881

**Published:** 2025-11-15

**Authors:** Sandeep Damaraju, Austin R Gomindes, Dylan Mistry, Pantelis Tsantanis, Govind S Chauhan

**Affiliations:** 1 Trauma and Orthopedics, University Hospitals Birmingham NHS Foundation Trust, Birmingham, GBR

**Keywords:** orthopedics and traumatology, posterior dislocation of the shoulder, shoulder injuries, shoulder scope, upper extremity trauma

## Abstract

Posterior shoulder dislocation is an uncommon condition, accounting for a minority of shoulder instability cases. It is more frequently encountered in high-level athletes and is often missed at initial presentation, which can lead to chronic instability and recurrent dislocations. Predisposing factors include congenital anatomical variations, capsular laxity, and acquired injuries such as trauma or tetanic muscle contractions during seizures or electrical shocks. Management strategies are broadly divided into conservative and surgical approaches. Nonoperative treatment focuses on strengthening the periscapular and rotator cuff musculature but has limited success in traumatic cases and in athletic patients, where long-term instability is more likely. Surgical options aim to address the underlying pathology and may be performed using open or arthroscopic techniques. Soft tissue procedures include labral repair and capsular plication, whereas bony procedures may involve corrective osteotomies in cases of congenital abnormalities or bone grafting to address reverse Hill-Sachs lesions resulting from humeral head impaction on the glenoid. We present a three-case series of posterior shoulder dislocations managed at a teaching hospital in the United Kingdom. This series highlights the spectrum of posterior shoulder instability, the multidisciplinary approach required, and the outcomes achieved with differing management strategies.

## Introduction

Posterior shoulder instability is a relatively uncommon condition, accounting for approximately 10% of all shoulder instability cases [1]. Its prevalence is higher among certain athletic populations, particularly those engaged in contact sports or repetitive overhead activities [2]. Because the condition is rare and patients often present with shoulder pain rather than classic instability symptoms, diagnosis is frequently delayed or missed [3]. Delays can also occur when initial presentations coincide with seizure management, further complicating timely recognition.

The mechanisms underlying posterior instability are diverse, ranging from high-energy trauma to more insidious causes. Traumatic posterior dislocations typically occur when the arm is flexed, adducted, and internally rotated under axial load. Repetitive microtrauma in this high-risk position can lead to progressive insufficiency of the capsule and labrum, particularly in athletes. Atraumatic cases may arise from capsular laxity, which can be idiopathic or related to underlying connective tissue disorders that compromise static stabilizers of the shoulder. Seizures and electrical shocks represent classic nontraumatic causes, during which violent contraction of the pectoralis major and latissimus dorsi forcibly drives the humeral head posteriorly [4].

Management strategies are broadly divided into conservative and surgical approaches. Nonoperative treatment focuses on strengthening the dynamic stabilizers of the glenohumeral joint, particularly the rotator cuff and periscapular musculature, and is effective for most low-demand patients with atraumatic instability [5]. However, conservative management has limited success in traumatic cases or in athletic individuals with high functional demands.

Surgical intervention may be necessary in these situations and can be performed via open or arthroscopic techniques. Because posterior instability does not result from a single pathological entity [4], careful preoperative assessment and multidisciplinary planning are essential for optimal outcomes. Operative strategies are generally categorized into soft tissue and bony procedures. Soft tissue techniques include capsular plication or shift, performed either open or arthroscopically, to reinforce the posterior capsule, the thinnest portion of the joint capsule, through medial or lateral advancement [6]. Bony procedures are indicated when osseous abnormalities contribute to instability and may involve corrective osteotomies for excessive glenoid retroversion or bone grafting to address posterior glenoid bone loss. Bone grafts, combined with capsular advancement, serve to restore stability throughout the shoulder’s arc of motion. Autografts are commonly harvested from the iliac crest, while allografts from femoral heads may also be used [7].

## Case presentation

We present a series of three cases of posterior shoulder instability, highlighting differing etiologies, a spectrum of management options, and the factors influencing clinical decision-making, including patient preferences, the limited existing evidence base, and the multidisciplinary approach required. Management considerations encompass the mechanism of injury, patient comorbidities, activity level, associated soft tissue or bony lesions, and the long-term goal of functional restoration.

Case 1: operative management

**Figure 1:**
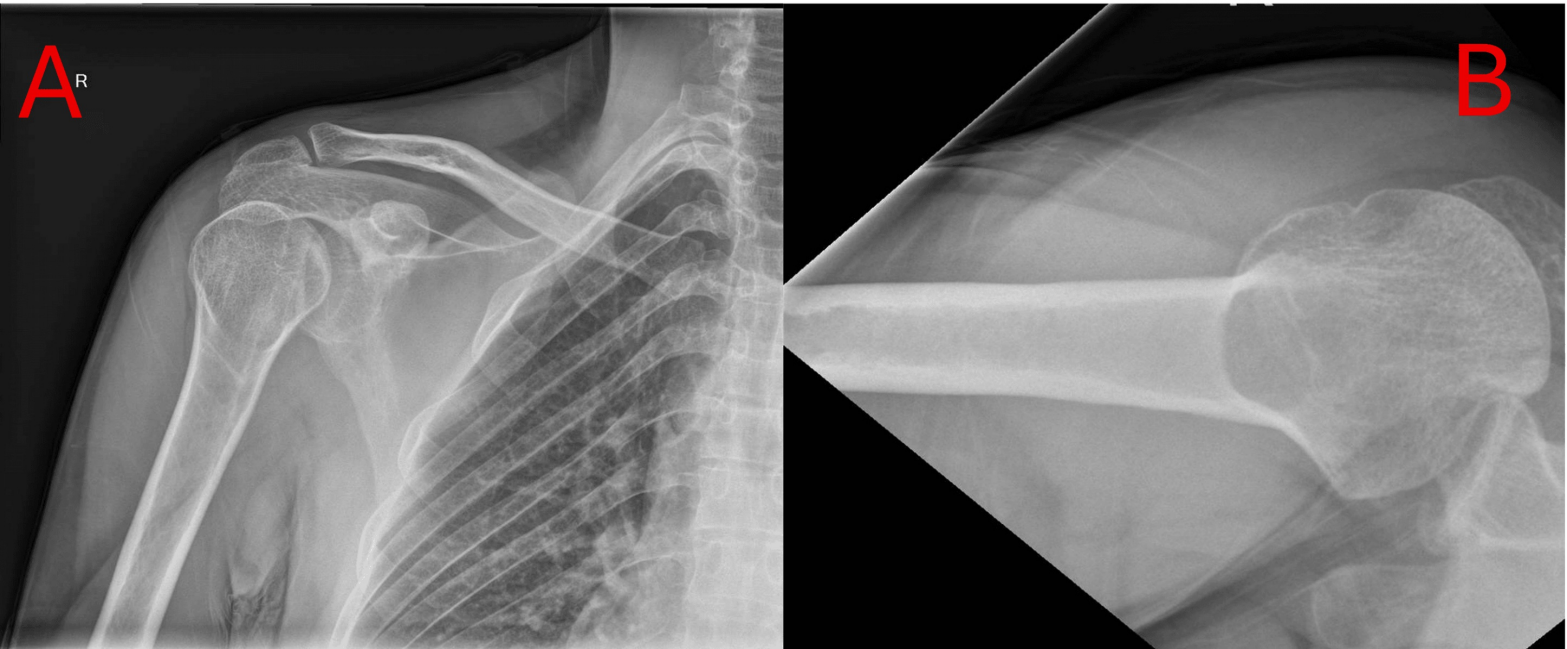
Plain AP radiograph (A) of the right shoulder, showing the classic “lightbulb” sign, and axillary view radiograph (B), demonstrating a large reverse Hill-Sachs lesion engaging the glenoid.

A 28-year-old man with a history of psychotic schizophrenia, managed with olanzapine and propranolol, and a background of smoking and illicit drug use, presented four months after an unprovoked seizure with his right shoulder locked in internal rotation. He was unable to achieve either active or passive range of motion but remained neurovascularly intact. There was no previous history of shoulder injury or instability. Radiographs obtained in the ED are shown in Figure 1.

Closed reduction under sedation in the ED was unsuccessful. The case was discussed at the upper-limb multidisciplinary team (MDT), and, given the chronicity of the dislocation, an urgent neurological review was obtained prior to surgery. Medical management was optimized to prevent further seizures. The patient was deemed fit for operative management and underwent open reduction with a modified McLaughlin procedure via a deltopectoral approach.

**Figure 2:**
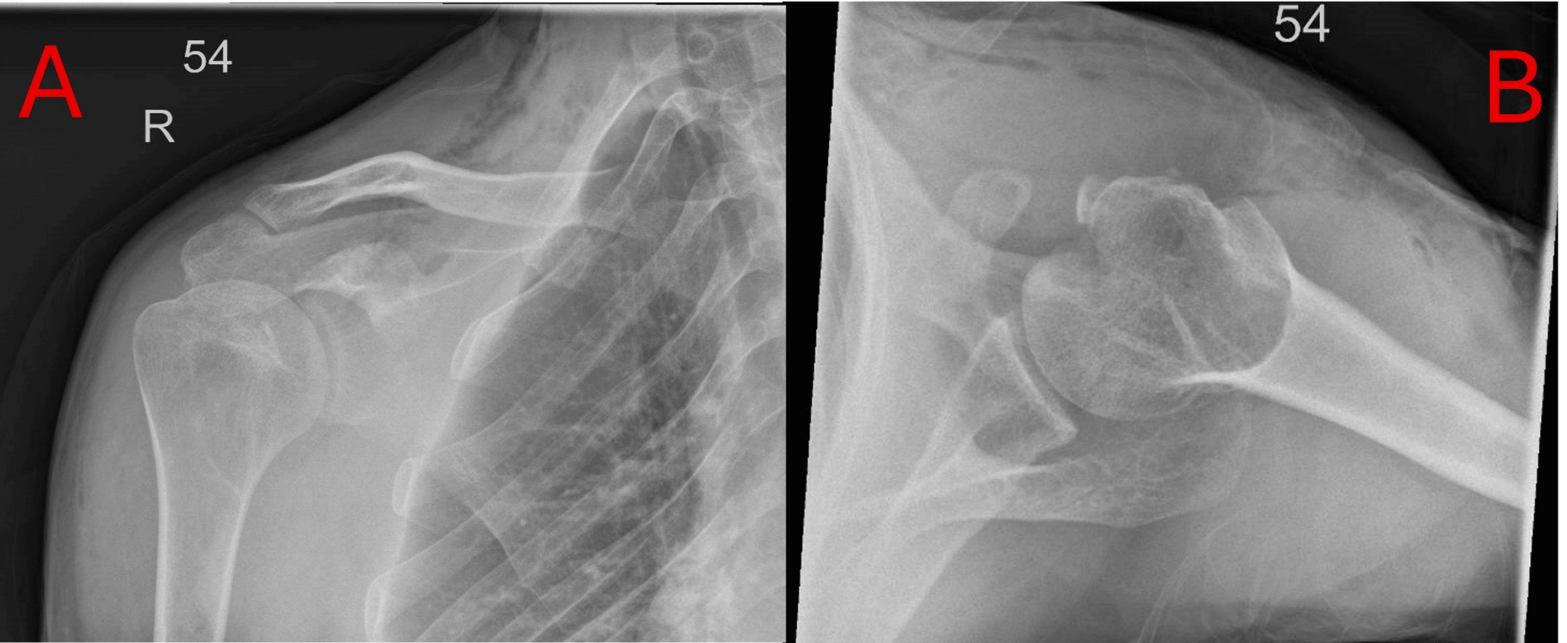
Plain postoperative AP radiograph (A) and axillary view radiograph (B) of the right shoulder, showing the humeral head appropriately positioned within the glenohumeral joint.

Intraoperatively, the humeral head was reduced, revealing a 3.0 × 1.5 cm reverse Hill-Sachs defect. The lesser tuberosity was tagged with FiberWire (Arthrex, Inc., Naples, Florida, USA), osteotomized, and the subscapularis mobilized. After preparation of the defect with a burr, the lesser tuberosity and subscapularis tendon were fixed into the lesion using Q-Fix anchors (Smith & Nephew plc, Hertfordshire, UK). Post-reduction manipulation confirmed joint stability, and postoperative radiographs demonstrated appropriate seating of the humeral head (Figure 2).

Unfortunately, the patient did not attend subsequent follow-up appointments despite repeated attempts at contact; however, no further admissions were noted in his electronic patient record.

Case 2: operative management

**Figure 3:**
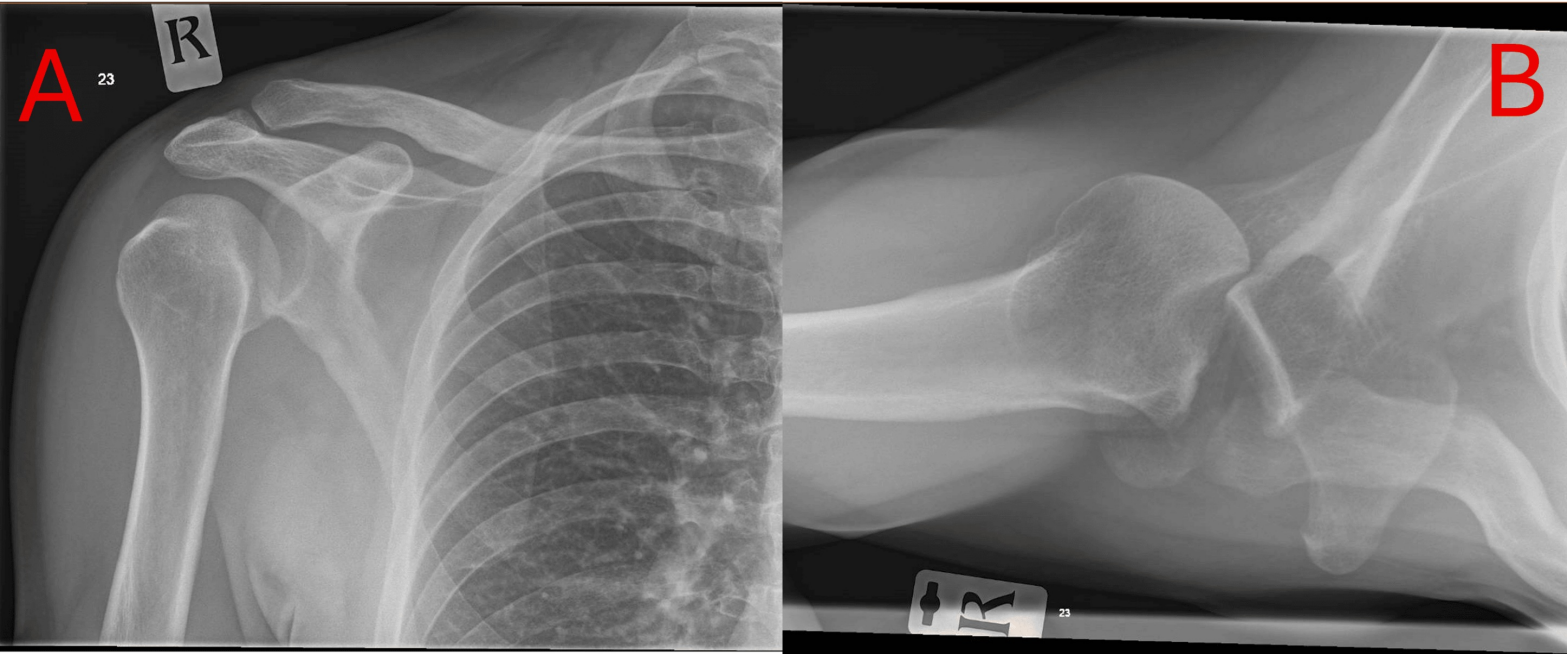
Plain AP radiograph (A) of the right shoulder and axillary view radiograph (B), showing a large reverse Hill-Sachs lesion engaging the glenoid.

A 37-year-old man sustained an atraumatic posterior dislocation of his right shoulder while showering in 2023. He had previously been investigated for seizures 10 years earlier but was not started on antiepileptic medication. He also has a history of excessive alcohol consumption. The patient reported a possible prior dislocation following a motorbike road traffic collision years earlier but did not seek medical attention at that time. He was initially managed with manipulation under sedation in the ED, with reduction confirmed radiographically (Figure 3), but he failed to attend follow-up for further evaluation and management planning.

Fifteen months later, he re-presented with another dislocation while showering, with no preceding seizure or trauma. Initial reduction under sedation was unsuccessful, so formal reduction under general anesthesia (GA) was performed in the theater. Following discussion of the risks and benefits, nonoperative management was pursued, and the patient was referred to physiotherapy.

Six months later, he experienced another dislocation while driving. Reduction under sedation in the ED was again unsuccessful, requiring formal reduction under GA. He was referred to the local Upper Limb Orthopedic service for consideration of operative management. CT imaging revealed a large reverse Hill-Sachs lesion. After discussion in the clinic, the patient opted for operative management but requested a four-month delay due to personal circumstances, acknowledging the risk of further instability.

Two months later, he sustained a traumatic posterior dislocation after a fall from standing height. Initial manipulation under sedation temporarily reduced the shoulder, but the joint immediately re-dislocated. The medical team was consulted to investigate the cause of the fall. The patient self-discharged against medical advice, accepting the risk of leaving the shoulder dislocated.

He returned five days later with severe pain. The shoulder was reduced under GA, and repeat CT scans confirmed a large reverse Hill-Sachs lesion similar to previous imaging. Medical review ruled out seizure activity; the patient was diagnosed with likely vasovagal episodes secondary to alcohol excess and dehydration, with no neurogenic cause identified.

One month later, the patient experienced another atraumatic dislocation, again requiring reduction under GA. At the MDT discussion, the consensus was to proceed with a modified McLaughlin procedure augmented with a femoral head allograft due to the defect size, the patient’s age, and his active lifestyle (manual labor and regular swimming).

**Figure 4:**
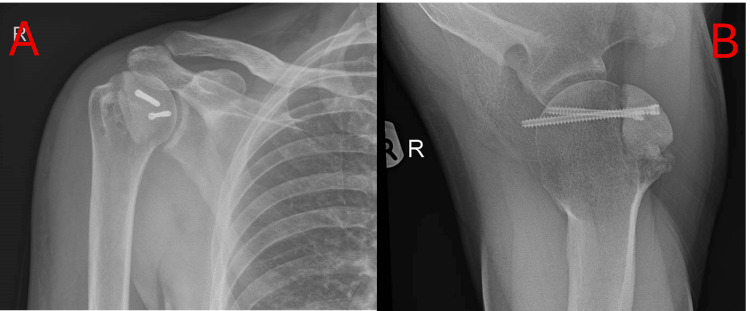
Plain postoperative AP radiograph (A) and axillary view radiograph (B) of the right shoulder, showing the humeral head within the glenohumeral joint and screw fixation of the allograft.

Prior to surgery, he sustained an additional dislocation, managed with manipulation under anesthesia. Intraoperatively, a 5 cm defect was identified (2.5 cm deep medially and 1.5 cm laterally). The defect was filled with a femoral head allograft and secured with two Medartis headless compression screws (Medartis AG, Basel, Switzerland). Subscapularis repair and biceps tenodesis were also performed. The patient was placed in an abduction sling for six weeks. Early postoperative clinic review demonstrated satisfactory wound healing with the joint remaining reduced (Figure 4), with further follow-up planned.

Case 3: nonoperative management

**Figure 5:**
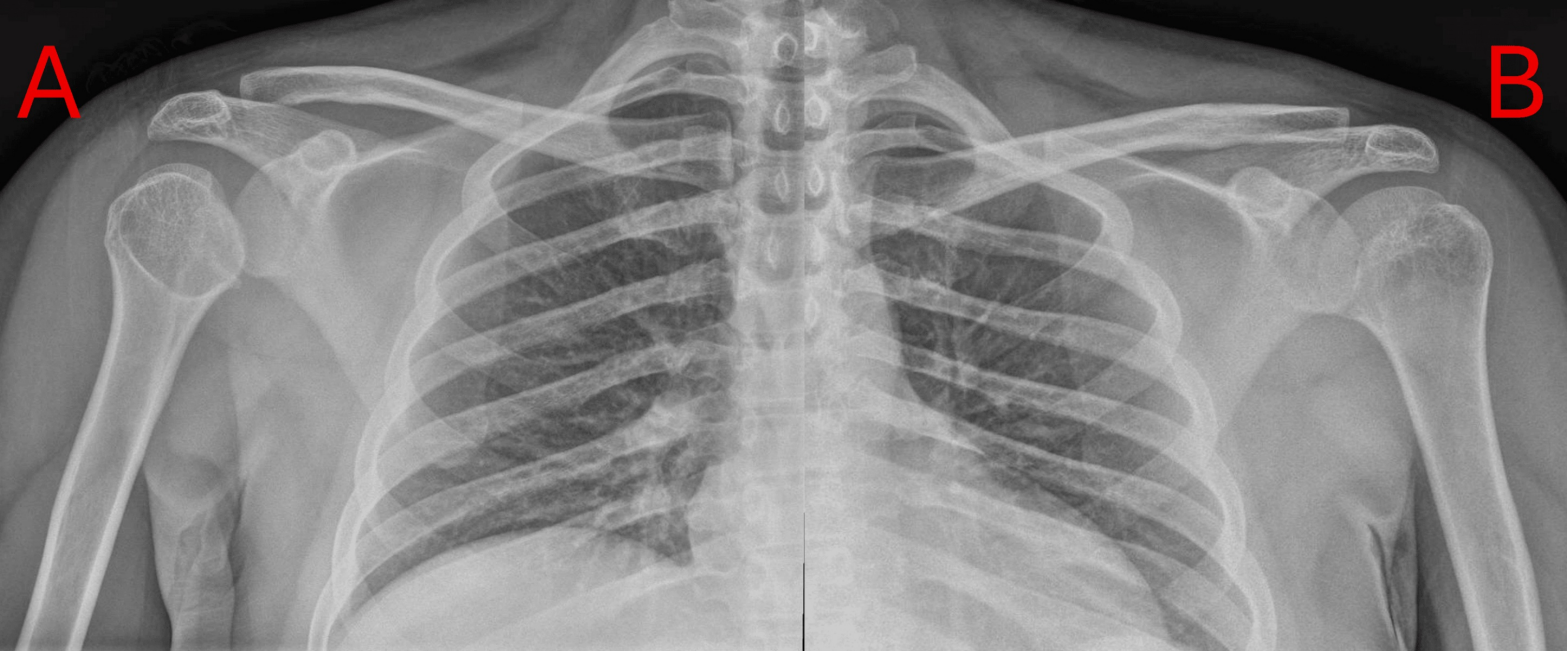
Plain AP radiographs of the right (A) and left (B) shoulders. The right shoulder shows the classic “lightbulb” sign.

A 33-year-old physiotherapist with a history of epilepsy and type 1 diabetes presented following a seizure secondary to a missed dose of medication. He sustained suspected bilateral posterior shoulder dislocations based on clinical history and subsequent imaging, with the left shoulder spontaneously reducing and the right shoulder relocated under sedation in the ED. Initial imaging is shown in Figure 5.

**Figure 6:**
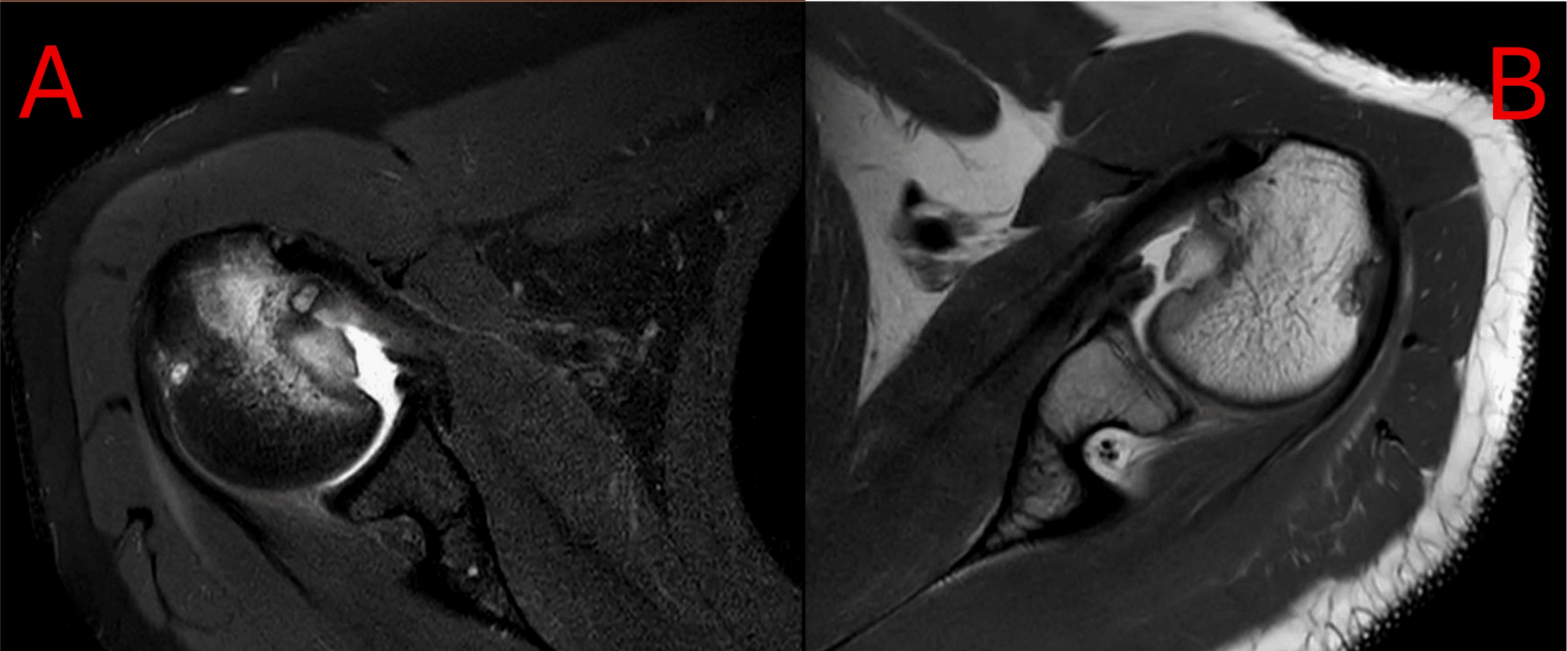
Transverse spectral adiabatic inversion recovery series MRI of the right (A) and left (B) shoulders, showing large reverse Hill-Sachs lesions.

MRI demonstrated a large reverse Hill-Sachs lesion on the right (3.0 × 3.3 cm) and a smaller lesion on the left (3.0 × 2.6 cm), as shown in Figure 6. On clinical examination, he retained nearly a full range of motion but had a positive apprehension test bilaterally (right worse than left). His primary concern was returning to work without symptoms.

After further discussion with the patient and the MDT, nonoperative management was pursued, alongside neurology input to optimize seizure control. At two months, the patient reported stiffness in the right shoulder, with complete loss of external rotation from neutral in the right shoulder and 10 degrees in the left; all other shoulder movements were preserved. The decision was made to continue with nonoperative management.

At six months, external rotation of the right shoulder had improved, although the patient continued to experience apprehension on provocation testing. Surgical intervention was discussed again, but the patient elected to defer any decision.

Telephonic consultation at 10 months demonstrated promising improvement in range of motion and reduction of pain, allowing the patient to perform all activities of daily living without restriction. Further in-person review was planned for clinical assessment.

## Discussion

Nonoperative management

Until recently, there has been limited evidence guiding the nonoperative management of posterior shoulder instability. Some studies suggest that conservative treatment is commonly employed, particularly in elderly or low-demand patients, where outcomes are generally favorable [3,8]. Rehabilitation strategies typically focus on strengthening the periscapular and rotator cuff musculature. In-season athletes may also benefit from this approach, as it can facilitate a quicker return to sport [9].

However, in highly active individuals, such as athletes and military personnel, nonoperative management has a high rate of failure, with many patients ultimately requiring surgical intervention [9]. A military cohort study sought to establish criteria for failure of conservative treatment [10]. It reported that patients whose primary complaint was pain were more likely to succeed with rehabilitation alone, whereas those with instability symptoms, particularly apprehension during overhead activities, were less likely to benefit and should be considered for operative management. Additionally, the presence of associated soft tissue injury or anatomical predisposition to instability was identified as a predictor of poor response to nonoperative treatment.

Operative management

Surgical treatment of posterior instability encompasses a heterogeneous group of procedures, tailored to the patient’s clinical presentation, anatomical pathology, and imaging findings. In our cases, modified McLaughlin procedures were selected due to the chronicity of the dislocations and the presence of large reverse Hill-Sachs lesions, where preservation of bone stock and restoration of functional activity were key priorities.

The literature demonstrates that posterior dislocations are frequently associated with humeral head bone loss [11]. When sufficiently large, reverse Hill-Sachs lesions can engage with the glenoid, producing recurrent instability even in the presence of intact soft tissue stabilizers [12]. Various surgical techniques have been described to address this pathology. Use of the subscapularis tendon, with or without lesser tuberosity transfer, to fill the defect is well established and has shown good outcomes in improving pain and stability [12,13]. Although some loss of external rotation may occur following this procedure, it is generally not functionally significant.

Reconstruction of the humeral head was first described by McLaughlin [14], who emphasized the importance of restoring the glenohumeral joint to prevent engagement of large reverse Hill-Sachs defects with the posterior glenoid. His original technique involved transferring the subscapularis tendon from the lesser tuberosity into the defect. This has since been modified to include transfer of the lesser tuberosity itself, providing improved bony filling of larger defects and further reducing the risk of engagement with the glenoid. Castagna et al. [15] reported that the modified McLaughlin procedure produced favorable medium- and long-term outcomes in patients with reverse Hill-Sachs lesions involving up to 50% of the humeral head.

However, concerns remain regarding adequate bony restoration in lesions approaching this threshold. To address this, several authors have described the use of structural bone grafts, both autografts and allografts, for humeral head reconstruction. Diklic et al. [16] presented a case series using femoral head allografts, demonstrating excellent postoperative outcomes and no recurrence of instability. Their work informed the operative approach used in our second case.

Limited evidence

While robust evidence exists to guide the management of anterior shoulder instability, including recent guidance from the British Elbow and Shoulder Society [17], the same cannot be said for posterior instability. The literature on these cases remains sparse, and there are currently no widely accepted, clinically applicable guidelines to support decision-making.

This lack of published evidence is partly attributable to the comparative rarity of posterior instability and its variable clinical presentation. Unlike anterior dislocations, which often present dramatically, posterior instability exists on a spectrum ranging from subtle, atraumatic symptoms of pain to frank dislocation. These factors contribute to delayed diagnosis, underreporting, and the consequent lack of high-quality studies.

Our series aims to contribute to this limited body of evidence by describing a range of presentations and management strategies. We emphasize the importance of tailoring treatment to patient-specific factors.

## Conclusions

Although posterior shoulder instability accounts for only a minority of instability cases, it represents a clinically significant pathology with the potential to substantially impair patient function. Compared with anterior instability, there is a relative paucity of literature, reflecting both the rarity of this condition and the diagnostic challenges it presents. This case series illustrates the complexity of management decisions and the spectrum of available treatment strategies. It underscores the importance of thorough patient counseling, multidisciplinary planning, and precise surgical technique where indicated. Management should always be tailored to the individual’s functional demands. While nonoperative treatment may achieve satisfactory results in lower-demand patients, its role is limited in highly active populations. Nevertheless, it may be considered if the patient wishes to avoid surgical risk, highlighting the importance of shared decision-making. Conversely, operative intervention remains the conventionally recommended approach when significant underlying pathology, such as bony defects or soft tissue injuries, is present. These interventions can provide excellent outcomes when carefully selected for the appropriate patient cohort.

Our cases contribute to the limited body of evidence on posterior shoulder instability and highlight the need for further studies to better define optimal management strategies. Obvious limitations exist in our report: only three cases are discussed, and follow-up for some patients is incomplete, limiting the generalizability of our findings. This underscores the need for large, multicenter studies on posterior instability to establish clear treatment guidelines.
